# Syndrome Differentiation Analysis on Mars500 Data of Traditional Chinese Medicine

**DOI:** 10.1155/2015/125736

**Published:** 2015-10-01

**Authors:** Yong-Zhi Li, Guo-Zheng Li, Jian-Yi Gao, Zhi-Feng Zhang, Quan-Chun Fan, Jia-Tuo Xu, Gui-E Bai, Kai-Xian Chen, Hong-Zhi Shi, Sheng Sun, Yu Liu, Feng-Feng Shao, Tao Mi, Xin-Hong Jia, Shuang Zhao, Jia-Chang Chen, Jun-Lian Liu, Yu-Meng Guo, Li Ping Tu

**Affiliations:** ^1^China Astronaut Research and Training Center, Beijing 100094, China; ^2^Data Center of Traditional Chinese Medicine, China Academy of Chinese Medicine Science, Beijing 100700, China; ^3^Shanghai University of Traditional Chinese Medicine, Shanghai 201203, China; ^4^Department of Control Science and Engineering, Tongji University, Shanghai 201804, China; ^5^Shanghai Daosheng Medical Technology Co. Ltd., Shanghai 201203, China

## Abstract

Mars500 study was a psychological and physiological isolation experiment conducted by Russia, the European Space Agency, and China, in preparation for an unspecified future manned spaceflight to the planet Mars. Its intention was to yield valuable psychological and medical data on the effects of the planned long-term deep space mission. In this paper, we present data mining methods to mine medical data collected from the crew consisting of six spaceman volunteers. The synthesis of the four diagnostic methods of TCM, inspection, listening, inquiry, and palpation, is used in our syndrome differentiation. We adopt statistics method to describe the syndrome factor regular pattern of spaceman volunteers. Hybrid optimization based multilabel (HOML) is used as feature selection method and multilabel *k*-nearest neighbors (ML-KNN) is applied. According to the syndrome factor statistical result, we find that qi deficiency is a base syndrome pattern throughout the entire experiment process and, at the same time, there are different associated syndromes such as liver depression, spleen deficiency, dampness stagnancy, and yin deficiency, due to differences of individual situation. With feature selection, we screen out ten key factors which are essential to syndrome differentiation in TCM. The average precision of multilabel classification model reaches 80%.

## 1. Introduction

With the development of the three-phase strategy, our manned space programme entered a new manned space station construction stage. How spaceman adapts to longtime isolation environment and overcomes the challenges from the aspects of body, mind, and spirit became a burning question in the area of manned space [1].

Mars500 mission was a psychology and physiology isolation experiment conducted by Russia, the European Space Agency, and China, in preparation for an unspecified future manned spaceflight to the planet Mars. A total of 640 experiment days were scheduled between 2007 and 2011, divided into three stages of differing length. During each stage, the crew of volunteers lived and worked in a mockup spacecraft. Communication with outside world was limited, and it was conducted with a realistic time delay of up to 25 minutes, to simulate the real-life communications lag between Mars and Earth. The final stage of the experiment was intended to simulate a 520-day manned mission. The mission was intended to yield valuable psychological and medical data on the effects of the planned long-term deep space mission. The experiment permitted the study of the technical challenges, work capability of crew, and management of long-distance spaceflight. Communications lag, autonomy, resource rationing, health, conditions of isolation, and hermetically closed, confined environment are the main peculiarities of the Martian flight.

As a complete medical system, TCM plays an indispensable role in medical care in China. Different from the reductionism thinking mode of western medicine, TCM is based on the holistic and systematic ideas. TCM practices are believed to be effective by many patients and scientists, sometimes offering palliative efficiency, while the practices of western medicine fail or are unable to provide treatment. We have reason to believe that TCM can play an important role in health security mission in long-term space flight.

In Mars500 mission, inspection, inquiry, and palpation of TCM were applied to study the state of human life activities in longtime isolation environment and to interpret the features and change rules. In this research, digital instrument was used to collect TCM diagnostic information of the spaceman volunteers. The scale of syndrome and symptom was designed to quantize the degree syndrome and symptom. Then, we got the digital and normalized information which was used to find the relationship between the symptoms and syndromes.

In our research, we apply statistics method to describe the syndrome factor regular pattern and find that qi deficiency is a base syndrome pattern throughout the entire experiment process. At the same time, there are different associated syndromes such as liver depression, spleen deficiency, dampness stagnancy, and yin deficiency, due to the differences of individual situation. Then, we search the objective and inherent relationship between the symptoms and syndromes.

In clinical practice, the relationship between symptoms and syndromes can be seen as multilabel classification problem in which many symptoms may present various syndromes. Many researches have been down by using multilabel learning in biomedical feild [2–6]. In our work, hybrid optimization based multilabel (HOML) [7] is used to select related features, and multilabel *k*-nearest neighbors (ML-KNN) [8] is applied as the multilabel classifier. In our model, ten important symptoms for syndrome differentiation are selected and they are all from inspection which includes complexion and tongue diagnosis. Then, we analyze the characteristics of complexion and tongue picture, finding that the changes of complexion and tongue picture are consistent with changes of syndromes.

The remaining of the paper is organized as follows: in Section 2, we introduce the data collection and preprocessing methods, the feature selection HOML, and the ML-KNN; we give the results and discussions of our research in Section 3; then, we make a conclusion.

## 2. Methods

Data collection, preprocessing, and data features TCM interpretation and software analysis were made before we got the dataset. The details are as follows.

### 2.1. Data Collection

According to the scheme of the TCM research of human body in Mars500 longtime isolation environment, DS01-T and auxiliary diagnosis system were used to collect TCM data from six spaceman volunteers every two weeks from June 3, 2010, to November 4, 2011. Inquiry, inspection (complexion and tongue picture), and palpation data of the spaceman volunteers are collected. This work is sponsored by China Astronaut Research and Training Center and all investigators signed the informed consent.

### 2.2. Data Preprocessing

The collected data were preprocessed and the ones meeting the requirement were stored in the database.

#### 2.2.1. Inquiry Data Preprocessing

Inquiry data in the scale of the inquiry were selected and united as the clinical terms by the panel of the TCM. The invalid data caused by mistakes of eyes or writing were eliminated. For example, the choice should be “before meals,” but the spaceman volunteers selected “after meals” instead. The same case may also happen to “daytime” and “night.” These mistakes were caused by writing obviously and were eliminated directly. There were also some logical conflicting mistakes. For example, the “bulimia” and “loss of appetite” may be selected at the same time. These mistakes were modified by eliminating one of the options according to the analysis of TCM experts.

#### 2.2.2. Inspection Data Preprocessing

The invalid data in the inspection data caused by accident were removed, such as blur pictures caused by the failure of the camera focusing or the shake of volunteers' tongues.

#### 2.2.3. Palpation Data Preprocessing

Palpation data were rectified by the panel of the TCM experts and the invalid ones caused by incorrect installation of sensor or sudden shake of volunteers were removed. The mistaken data contained the pulse information which could not be recognized by software and the experts or the signal that results from main peak of the pulse was less than 10 mmHg.

### 2.3. TCM Interpretation and Software Analysis of Data Features

Interpretation of the TCM experts and analysis of the software were introduced to the interpretation and analysis of data features. The details can be as follows.

#### 2.3.1. TCM Experts Interpretation of Data Features

The panel of TCM consisted of three chief physicians whose clinical experience was over 20 years. Three experts worked alone at first and then compared their results. Results would be obtained as final ones when their results were consistent. Otherwise, the final results would be made by the panel of TCM discussion with the other three TCM experts:

- interpretation of inspection data: interpretation of inspection data was generated from analysis of tongue and facial pictures; then, the qualitative description and possible medical significance of tongue and facial features were given;
- interpretation of palpation data: palpation pictures of spaceman volunteers were analyzed to generate the interpretation of palpation data; the information of volunteers' palpation pictures, such as pulse position, pulse rate, pulse power, rhythm, and pulse shape was analyzed combined with common pulse condition model in the former database; the qualitative interpretation and the possible medical significance of the pulse condition features were given and used as one basis of the syndrome differentiation;
- interpretation of inquiry data: the descriptions of part of symptom in the syndrome and symptom scale were translated into standard terminology; the symptom was regarded as main symptom or general symptom by TCM experts according to the frequency and degree of the symptom and the clinical experience; then, inquiry results were interpreted to analyze volunteers' health condition;
- interpretation of syndrome: analysis of syndrome was based on the information fusion of the inquiry, complexion, and tongue picture and pulse condition.

#### 2.3.2. Software Analysis of the Data Features

**Figure 1:**
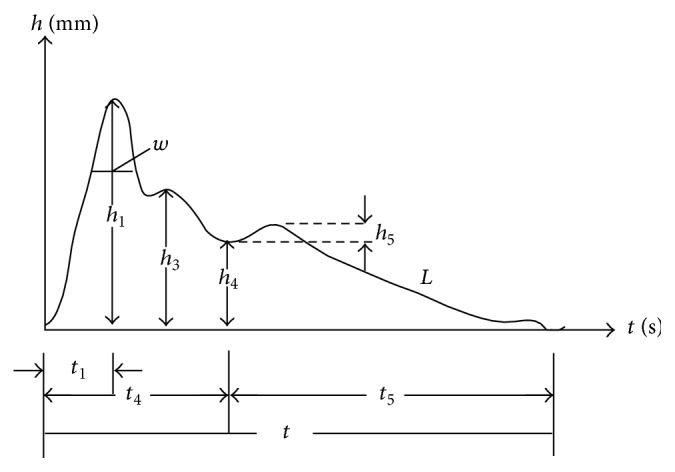
Basic structure of pulse picture.

(i) Interpretation of tongue picture and complexion features: interpretation of tongue picture and complexion features generated from analysis software is listed in Table 1. Results from analysis software should be considered with the ones from TCM experts.
(ii) Interpretation of pulse condition features: pulse pictures were obtained from palpation data.  Figure 1 presents the basic structure of pulse picture. Relationship between amplitude and phase of pulse wave was analyzed by software using time-domain analyzing method. The analysis content contained the recognition of height of wave and gorge, the corresponding value, and the area of the pulse picture. Notations in Figure 1 can be interpreted as follows:
   
*h*_1_: amplitude of the main wave,
   
*h*_3_: front wave amplitude of dicrotic pulse,
   
*h*_4_: amplitude of dicrotic notch,
   
*h*_5_: amplitude of dicrotic pulse,
   
*t*_1_: acute ejection period value,
   
*t*_4_: systole value,
   
*t*_5_: diastole value,
   
*t*: pulsation period,
   
*w*: one-third of *h*_1_.

Then, the features of pulse condition were interpreted by using frequency-domain analysis and time-frequency analysis, based on principles of hemodynamic methods.

### 2.4. Dataset Description

Through above preprocessing, we get a data set with 222 cases in which each case has 389 features and 11 labels. The inspection data contains 245 features, the palpation data contains 30 features, and the inquiry data have 114 features.

### 2.5. Feature Selection

In TCM diagnosis, a patient may be associated with more than one symptom, and its computer-aided diagnosis is a typical application in the domain of multilabel learning of high-dimensional data. It is common that a great deal of symptoms can occur in TCM diagnosis, which affects the modeling of diagnostic algorithm. Feature selection entails choosing the smallest feature subset of relevant symptoms and maximizing the generalization performance of the model. In this work, HOML is used to analyze feature selection for multilabel TCM data. HOML combines the relatively strong global optimization ability of simulated annealing algorithm (SA) [9], genetic algorithm (GA) [10], and the strong local optimization capability of greedy algorithm [11]. The following is the details of HOML, which organizes a search in three stages.

*Stage 1*. A simulated annealing (SA) is employed to guide the global search in a solution space. SA would accept every solution if the temperature is very high, which then yields a near random search through the search space. As the temperature becomes close to zero, only improvements are accepted. The SA is run for approximately 50% of the total time available.

*Stage 2*. A GA is employed to perform optimization. The GA population is set at 100. The initial population consists of the best solutions detected by SA. The crossover operator enables the good solutions to exchange information, and the mutation operator in GA introduces new genes into the population and retains genetic diversity. The GA runs for about 30% of total time spent by HOML to find the optimal feature subset solution.

*Stage 3*. A hill-climbing feature selection algorithm is applied. The greedy algorithm performs a local search on the *k*-best solutions on the *k*-best (*k* represents the dimensionality of feature) solutions given by two global optimization algorithms (SA and GA).

### 2.6. Multilabel Classifier

In our study, the multilabel *k*-nearest neighbour (ML-KNN) algorithm is used to analyse syndromes models. KNN is an algorithm whose idea is to search for the nearest point in training dataset [12]. In KNN algorithm, an instance is regarded as a point. And the label of a test instance is probably similar to that of several nearest points. Based on this theory, the algorithm of KNN is to search for *k* train instances nearest to the test instance, then according to their labels, to predict the label of the test instance. Compared with other mining algorithms, the advantage of KNN lies in simpler training process, better efficiency, and forecast accuracy.

In the multilabel data, just simple splitting may result in data loss because of the relationship between each label. At this condition, multilabel KNN would be a better choice to solve this problem. ML-KNN is the first multilabel lazy learning algorithm, which is derived from the popular *k*-nearest neighbor (KNN) algorithm. The basic idea of ML-KNN is to adapt *k*-nearest neighbor techniques to deal with multilabel data, where maximum a posteriori (MAP) rule is applied to make prediction with the labeling information embodied in the neighbors [13]. In a word, the labels of each instance are judged by its nearest neighbors. Brief introduction of this algorithm is shown as follows.

**Step 1 .**

The conditional probability distribution between each instance and its associated label set would be calculated at first.

**Step 2 .**

Calculate the distance between each test instance and the training instances; then find *k*-nearest instances for each test instance.

**Step 3 .**

For each test instance, its forecast results would be acquired according to the labels of *k*-nearest training instances and the conditional probability associated to each label.

**Step 4 .**

Evaluate the forecast results according to multilabel evaluation criteria.

### 2.7. Experimental Design and Evaluation

In our experiment, 5-fold cross-validation is utilized to test the accuracy of the classification. We firstly build three classification models with four types of diagnostic fusion data, inspection data, and palpation data, respectively, by using ML-KNN. Then, we apply HOML to the model which obtains the best performance.

Let *X* denote the domain of instances and let *Y* = {1,2,…, *Q*} be the finite set of labels. The multilabel classification problem can be formulated as follows. Given a training set *T* = {(*x*_1_, *Y*_1_), (*x*_2_, *Y*_2_),…, (*x*_*m*_, *Y*_*m*_)}  (*x*_*i*_ ∈ *X*, *Y*_*i*_ ∈ *Y*), drawn from an unknown distribution D, the goal of the learning system is to output a multilabel classifier *h* : *X* → 2^*Y*^ which optimizes some predefined criteria. The learning system will tend to output larger values for labels in *Y*_*i*_ than those which are not in *Y*_*i*_ according to a real-valued function of the form *X* × *Y* → *R*. For example, if *y*_1_ ∈ *Y*_*i*_ and *y*_2_ ∉ *Y*_*i*_, then *f*(*x*_*i*_, *Y*_1_) > *f*(*x*_2_, *Y*_2_).

A ranking function rank_*f*_(·, ·), which can be the transformed form of the real-valued function *f*(·, ·), maps the outputs of *f*(*x*_*i*_, *y*) for any *y* ∈ *𝕐* to {1,2,…, *Q*}. For example, for the *f*(*x*_*i*_, *y*_1_) > *f*(*x*_2_, *y*_2_), there will be rank_*f*_(*x*_*i*_, *y*_1_) < rank_*f*_(*x*_*i*_, *y*_2_). Then, the multilabel classifier *h*(·) can be represented as *h*(*x*_*i*_) = *y*∣*f*(*x*_*i*_, *y*) > *t*(*x*_*i*_), *y* ∈ *Y*, in which *t*(·) is a threshold function.

It is worth noting that in multilabel learning paradigm, various evaluation criteria have been proposed to measure the performance of a multilabel learning system. Given a test set *S* = {(*x*_1_, *Y*_1_), (*x*_2_, *Y*_2_),…, (*x*_*p*_, *Y*_*p*_)}, the following multilabel evaluation metrics are used in this paper [8].

(1) Hamming loss is defined as

(1) $$\begin{array}{c} {hloss}_{S}\left(h\right)=\frac{1}{p}\overset{p}{\underset{i=1}{\sum }}\frac{1}{Q}\left|h\left(x_{i}\right)\Delta Y_{i}\right|, \end{array}$$

where Δ stands for the symmetric difference between two sets. Note that when |*Y*_*i*_| = 1, for all instances, a multilabel system is in fact a multiclass single-label one and the hamming loss is 2/*Q* times the usual classification error. Hamming loss is used to evaluate how many times an instance-label pair is misclassified. The smaller the value of hloss_*S*_(*h*), the better the performance.

(2) One-error is defined as

(2) $$\begin{array}{c} {\text{one-error}}_{S}\left(f\right)=\frac{1}{p}\overset{p}{\underset{i=1}{\sum }}\left[\left[\left[\underset{y\in Y}{arg\;max}f\left(x_{i},y\right)\right]\notin Y_{i}\right]\right], \end{array}$$

where for any predicate *π*, [*π*] equals 1 if *π* holds and 0, otherwise. Note that, for single-label classification problems, the one-error is identical to ordinary classification error. One-error is used to evaluate how many times the top-ranked label is not in the set of proper labels of the instance. The smaller the value of one-error_*S*_(*f*), the better the performance.

(3) Ranking loss is defined as

(3) rlossSf=1p∑i=1p1YiYi¯y1,y2fxi,y1≤fxi,y2,Yi¯mmmmmmmmmmy1,y2∈Yi×Yi¯,

where $\bar{Y}$ denotes the complementary set of *Y* in *y*. Ranking loss is used to evaluate the average fraction of label pairs that are reversely ordered for the instance. The smaller the value of rloss_*S*_(*f*), the better the performance.

(4) Average precision is defined as

(4) avgprecSf =1p∑i=1p1Yi  ×∑y∈Yiy′ ∣ rankfxi,y′≤rankfxi,y,y′∈Yirankfxi,y

and is used to evaluate the average fraction of labels ranked above a particular label *y* ∈ *Y* which actually are in *Y*. The bigger the value of avgprec_*S*_(*f*), the better the performance.

## 3. Results and Discussion

### 3.1. Syndrome Factor Statistical Result

**Figure 2:**
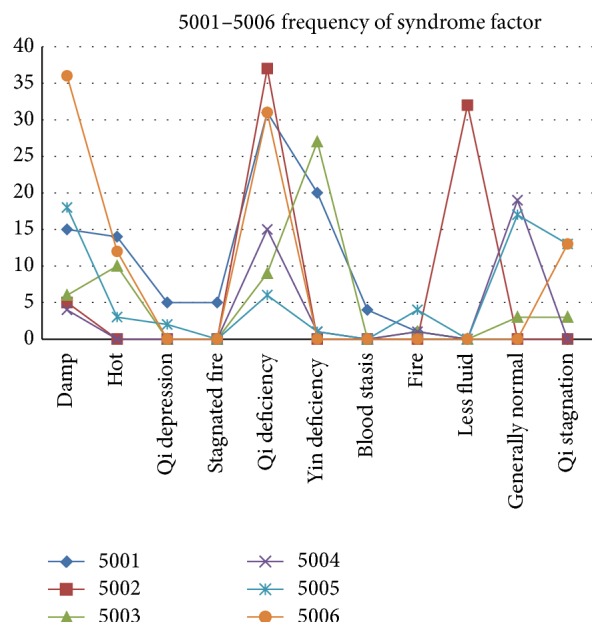
Frequency of syndrome factor.

In this section, statistics method is applied to describe the syndrome factor regular pattern and the result is shown in Figure 2. From Figure 2, we find that qi deficiency is a base syndrome pattern throughout the entire experiment process and, at the same time, there are different associated syndromes such as liver depression, spleen deficiency, dampness stagnancy, and yin deficiency, due to differences of individual situation.

### 3.2. Results by Using Multilabel Learning Methods

Results of ML-KNN without HOML are shown in Table 2 and results of ML-KNN with HOML are shown in Table 3. Comparing Tables 2 and 3, we find that ML-KNN with HOML obtains better performance than that without HOML which means that feature selection plays an important role in our model. Feature selection results of our model are shown in Table 4. As shown in Table 4, we can see that the ten important features selected are all in complexion and tongue diagnosis. Then, we analyze the characteristics of complexion and tongue picture in the following.

### 3.3. Characteristics of Complexion and Tongue Picture

#### 3.3.1. Change Characteristics of Tongue Picture Objective Indicators

After extraction of characteristic value of tongue picture, respectively, we calculate sample for tongue body and each part of coating on the tongue on Lab average value. The facial overall is the average value for each part and we select smooth quarter (3 months). Using them, we map the time trends figure. Because of the original material, we deal with the lack of four tongue images using nearest neighbor interpolation method in which it is the average value of before and after neighbors.

**Figure 3:**
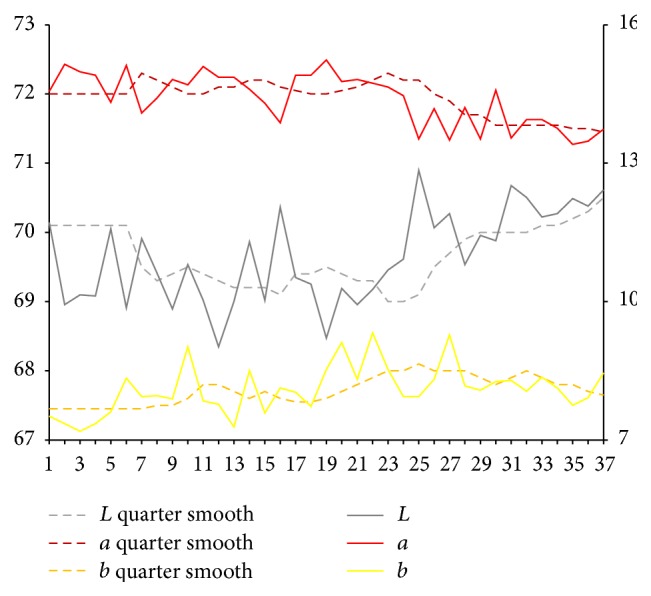
Lab value changing trend of tongue body.

**Figure 4:**
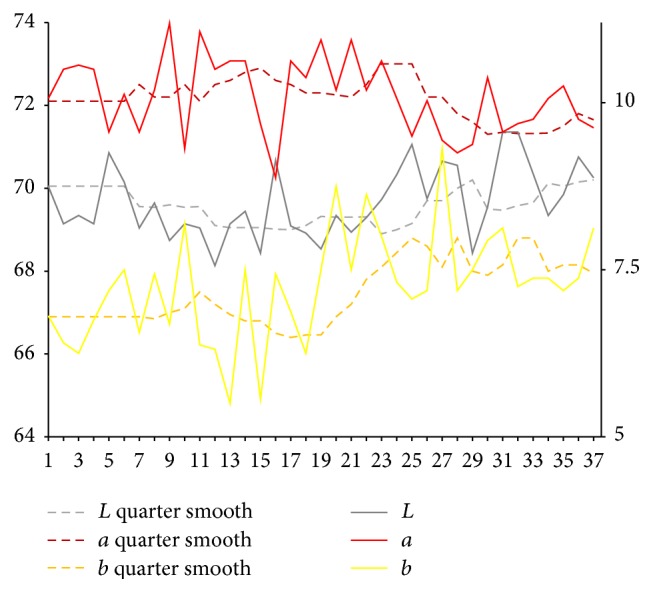
Lab value changing trend of coating on the tongue.

Results are shown in Figures 3 and 4. From Figures 3 and 4, it can be seen that, compared with the initial state, the brightness *L* values of tongue body and coating on the tongue first show a trend of decrease and later show a substantial increase. In the late (beginning from 25th), the brightness *L* values increase significantly. Early and midterm body of the tongue show a weakly rising, and, in the late (beginning from 25th), *a* values show a slight reduction. In the late (beginning from 20th), *b* value of coating on the tongue continues to rise significantly.

#### 3.3.2. Change Characteristics of Complexion Objective Indicators

After the extraction of characteristic value of complexion image, respectively, we calculate sample for each part of facial overall on Lab average value. The facial overall is the average value for each part and we select smooth quarter (3 months). Using them, we map the time trends figure. Because of the original material, we deal with the lack of three complexion images using nearest neighbor interpolation method in which it is the average value of before and after neighbors.

**Figure 5:**
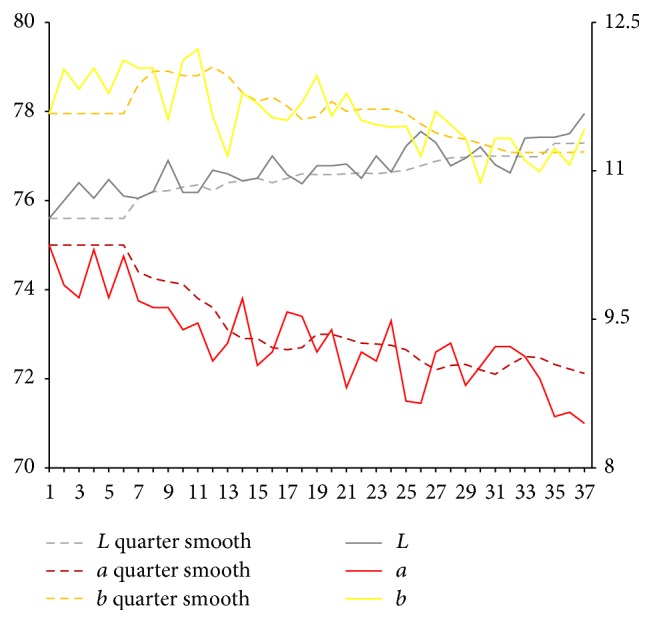
Lab value changing trend of facial overall.

Lab value changing trend of facial overall is shown in Figure 5. From Figure 5, it can be seen that, compared with the initial state, the brightness *L* value of facial overall first shows a trend of continued increase and *a* value shows a trend of continued decrease.

**Figure 6:**
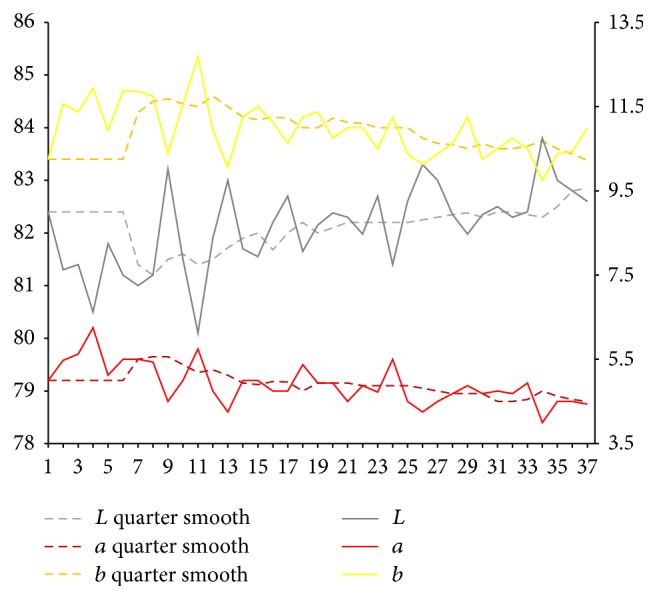
Lab value changing trend of forehead.

**Figure 7:**
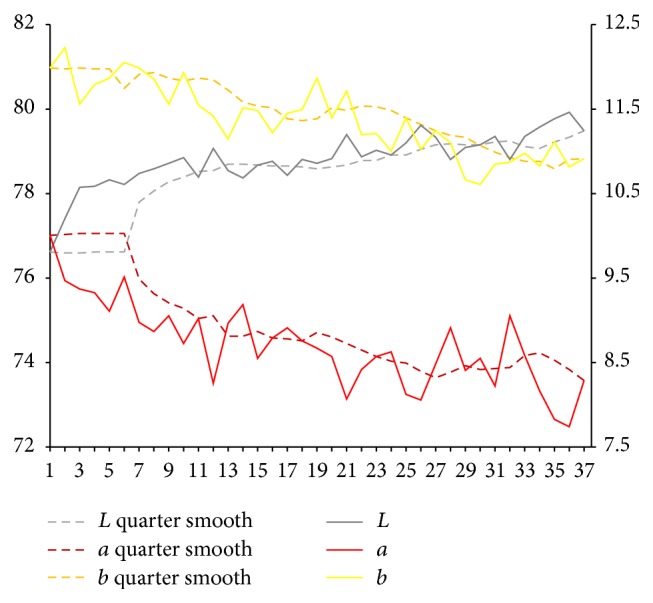
Lab value changing trend of nose.

**Figure 8:**
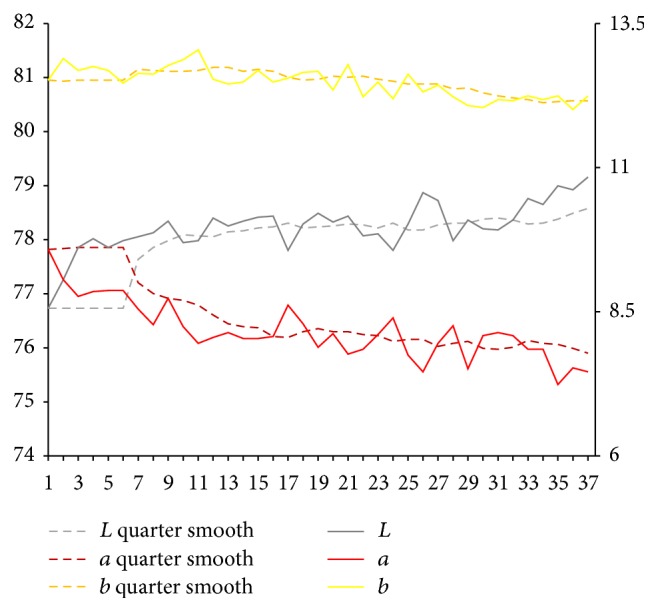
Lab value changing trend of left cheek.

**Figure 9:**
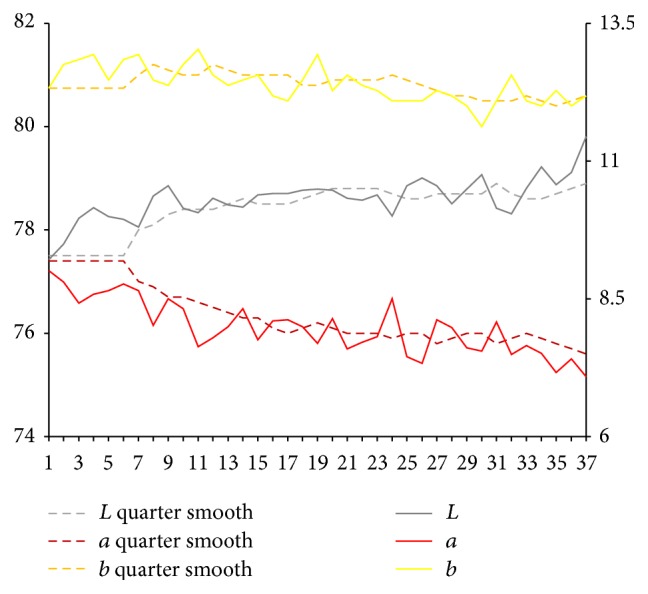
Lab value changing trend of right cheek.

**Figure 10:**
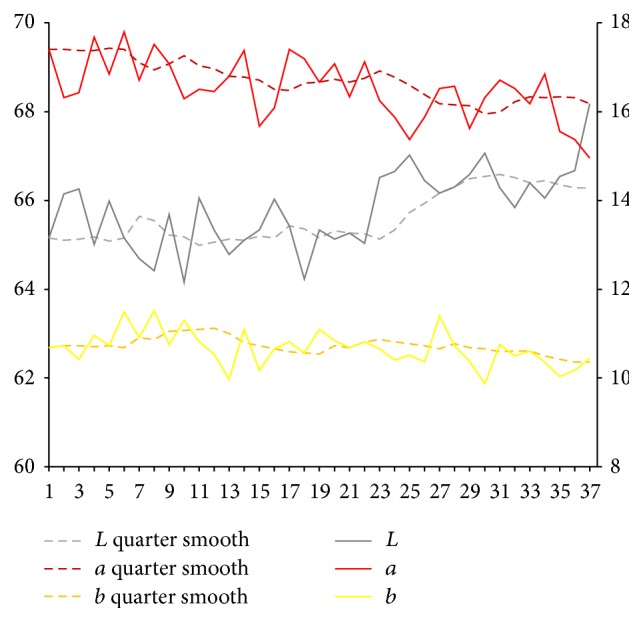
Lab value changing trend of lip.

**Figure 11:**
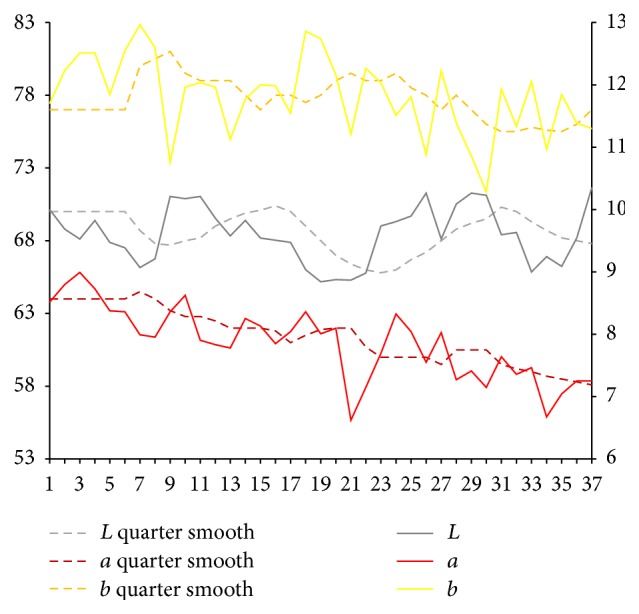
Lab value changing trend of underjaw.

From Figures 6, 7, 8, 9, 10, and 11, it can be seen that compared with the initial state, the brightness *L* values of forehead, nose, left cheek, right cheek, and lip show a trend of increase, in which forehead and nose are more obvious. Brightness *L* value of underjaw shows a cyclical change. Values of forehead, nose, left cheek, right cheek, lip, and underjaw show a slight reduction in different degree, in which nose is the most obvious.

#### 3.3.3. Analysis of Complexion and Tongue Picture Characteristics

**Figure 12:**
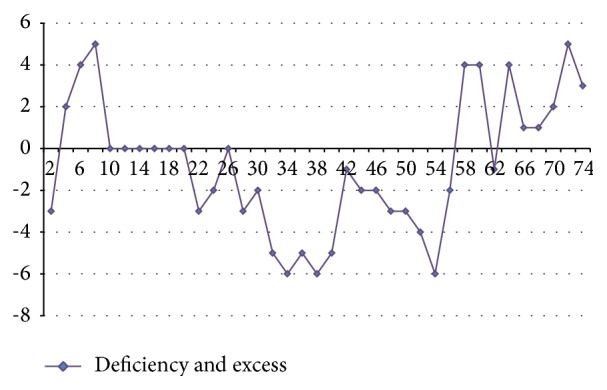
Variation of syndromes.

From the above results, it can be seen that, after spaceman volunteers get into airtight cabin, changes of tongue picture and complexion are consistent with changes of syndromes which are shown in Figure 12. Brightness *L* values of tongue body and coating on the tongue firstly show a trend of decrease, then increase, and begin to increase significantly since the 50th week. At early and middle stage of getting into cabin, *a* value of tongue body increases weakly, and, since the 50th week, *a* value tends to slightly decrease. Since the 40th week of getting into cabin, *b* value of coating on the tongue continues to increase significantly. Compared with the early stage of getting into cabin, brightness *L* value of facial overall continues to increase, and *a* value continues to decrease. Brightness *L* values of forehead, nose, left cheek, right cheek, and lip show an increasing trend, among which forehead and nose are more obvious and lip increases more obvious at late stage (since 50th). Brightness *L* value of underjaw changes periodically. Values of forehead, nose, left cheek, right cheek, and lip show decreasing trend in different degrees, and nose is most obvious, which indicates brightness *L* values and *a* values of tongue picture; complexion of spaceman volunteers has different degrees of volatility; prompting volunteers are in the adaptation state, and all syndromes are existent. After a period of time of getting into cabin especially since the 50th week, brightness *L* values of tongue picture and complexion gradually increase, while *a* values gradually decrease, which presents deficiency syndrome, and it is consistent with the changes of syndromes. Besides, in the isolation environment, body cannot be exposed to sunlight for long time, and there will be changes in color values of complexion, manifested as increasing brightness and decreasing red luminosity.

### 3.4. Analysis of Feature Selection Results of Our Model

In the mining process, we found that complexion was obvious in feature selection among the single index of complexion, pulse, and tongue. One reason is that changes of deficiency of qi and blood are before the change of pulse. Because of deficiency of qi and blood, complexion is easy to show up. The contractility, resistance, and tonicity are no response from pulse, because the deficiency of vital energy is weak and body is in good physical quality. There are no problems about cardiac systolic function and the appearance of peripheral resistance. On the characterization, mainly, color and pulse condition are not significantly affected. Red color is the reaction of tongue body and the thin white coating is the reaction of the coating on the tongue.

Qi deficiency is the main syndrome of spaceman volunteers in isolation environment. Tongue and complexion are the most sensitive among four diagnostic methods in qi deficiency.

Operation and system error also have a certain influence to the contribution of pulse. People can maybe do this, but machine has the certain difficulty.

## 4. Conclusions

In this paper, statistics method is adopted to describe the syndrome factor regular pattern, finding that qi deficiency is a base syndrome pattern throughout the entire experiment process. While there are different associated symptoms such as liver depression, spleen deficiency, dampness stagnancy, and yin deficiency, due to differences of individual situation, machine learning methods are applied to mine the relationship between symptoms and syndromes. In our work, HOML is used to selected related symptoms and ML-KNN is used as the multilabel classifier. Compared with the model without HOML, the model with HOML obtains better performance. Through feature selection, ten key symptoms are selected for syndrome differentiation. Then, we give a detailed discussion for the feature selection results. At the same time, the average precision of multilabel classification model reaches 80%.

In this research, our syndrome differentiation results reveal base syndrome features and evolvement rule for human body in longtime isolation environment, which lays the foundation for further research. In the next work, we would do much research on how to improve the classification accuracy and, with higher classification accuracy, the multilabel classification model can aid decision making for syndrome differentiation.

## Figures and Tables

**Table 1:** Qualitative and quantitative features of inspection.

Parameter	Qualitative features	Quantitative features
Tongue color	Dark, pale red, red, crimson, pale purple, dark purple	RGB, HSV mean, feature color proportion
	Red tongue tip	Tongue tip RGB, HSV mean color difference between tongue tip and the rest

Fur color	White, yellow and white, yellow, and gray black	RGB, HSV mean, proportion of feature color

Texture of fur	Thick fur, thin fur	Density degree
	Curdy fur, greasy fur	Density degree, distribution
	Less moss	Coverage ratio
	No moss	Coverage ratio
	Peeling fur	Defect area

Tongue shape	Fat, thin	Degree of circularity, length-width ratio, with degree
	Fast insertion	Number and color of tongue tip's circle dot
	Crack	Proportion of crack area
	Indentation	Size of indentation area
	Petechia	Number and color of tongue tip's circle dot
	Ecchymosis	Ecchymosis position, ecchymosis RGB, HSV mean

Complexion	Blue, red, yellow, white, black, normal	RGB, HSV mean, feature color proportion
Gloss	Glossy, few gloss, no gloss	RGB, HSV mean, feature color proportion
Lip color	Dark, red, dark red, purple	RGB, HSV mean, feature color proportion

**Table 2:** Classification results without HOML.

Dataset	Average precision	Ranking loss	One error	Hamming loss
Four diagnostic fusion data	0.78	0.10	0.26	0.12
Inspection data	0.77	0.10	0.31	0.12
Palpation data	0.69	0.15	0.36	0.14

**Table 3:** Classification results with HOML.

Dataset	Average precision	Ranking loss	One error	Hamming loss
Four types of diagnostic fusion data	0.80	0.09	0.25	0.12

**Table 4:** Feature selection results by using HOML.

Dataset	Feature selection results
Four types of diagnostic fusion data	RGB_B_face, Lab_A_tongue_coating, HSV_S_face
	HSV_H_face, RGB_G_face, Lab_B_face, Lab_L_face
	RGB_R_face, HSV_V_face, Lab_B_tongue_coating
